# Clinical Features and Risk Factors for Mortality in Hospitalized Older Adults with Pneumonia

**DOI:** 10.1155/2021/5644824

**Published:** 2021-11-16

**Authors:** Nobuhiko Fukuda, Nobuaki Kobayashi, Makoto Masuda, Aya Wakabayashi, Nobuko Kusano, Keisuke Watanabe, Nobuyuki Horita, Yu Hara, Masanori Nishikawa, Takeshi Kaneko

**Affiliations:** ^1^Department of Pulmonology, Yokohama City University Graduate School of Medicine, Yokohama, Japan; ^2^Department of Respiratory Medicine, Fujisawa City Municipal Hospital, Fujisawa, Japan

## Abstract

**Background:**

Pneumonia is a common disease among the aging population in Japan. Hence, it is important to elucidate the risks related to pneumonia mortality. Since *Streptococcus pneumoniae* is the most commonly observed pathogen, pneumococcal vaccination is recommended to older adults. Therefore, this study aimed to clarify the clinical features of pneumonia, including the status of pneumococcal vaccination, in hospitalized older adult patients in Japan.

**Methods:**

This single-centered retrospective study was conducted by reviewing the medical records of all patients with acute pneumonia at Fujisawa City Hospital in Japan from April 2018 to March 2019. Patients were divided into two groups based on their history of pneumococcal vaccination. The primary endpoint was in-hospital mortality, while the secondary endpoint was risk factors associated with mortality.

**Results:**

We included 93 patients with pneumonia in this retrospective study. Although the mortality rate was higher in the vaccinated group (15.8%) than in the unvaccinated group (9.1%), vaccination status was not identified as a significant risk factor for mortality after multivariable logistic regression (odds ratio: 2.71; 95% confidence interval: 0.667–11.02; *p*=0.16). In addition, the A-DROP score was identified as an independent risk factor (odds ratio: 2.64; 95% confidence interval: 1.22–5.72; *p*=0.008).

**Conclusions:**

Our study suggested that the A-DROP score is a risk factor of mortality for pneumonia in older adults. In addition, pneumococcal vaccination history was related to increased mortality; however, the influence of the vaccination remains unclear because of the small sample size.

## 1. Introduction

Pneumonia is a common disease among the aging population in Japan. Although there is rapid progress in its treatment, pneumonia remains one of the leading causes of death. In 2018, pneumonia (excluding aspiration pneumonitis) was the fifth most common cause of death in Japan, especially for older adults >65 years [1]. It is important to elucidate the risk factors for pneumonia related to death.

*Streptococcus pneumoniae* is the most common pneumonia pathogen. In Japan, the 23-valent pneumococcal polysaccharide vaccine (PPV23) and the 13-valent pneumococcal conjugate vaccine (PCV13) are available, and older adults >65 years can receive PPV23 at public expense every five years. Some clinical trials have investigated the effects of these vaccines in preventing pneumococcal pneumonia and reducing the risk of mortality [2–4]. In particular, the vaccine efficacy of PPV23 was shown to be 73% in preventing cases of invasive pneumococcal disease (IPD) [5]. However, it was reported that the effects of PPV23 and PCV13 were limited against nonbacteremic pneumonia [6–10]. This study aimed to clarify the clinical features of pneumonia in hospitalized older adult patients in Japan and investigated how pneumococcal vaccination was related to pneumonia. This study has been presented in accordance with the STROBE reporting checklist.

## 2. Materials and Methods

### 2.1. Study Design and Patient Selection

This retrospective study was conducted by reviewing data of all patients with acute pneumonia at Fujisawa City Hospital in Japan from April 2018 to March 2019. We included patients with a diagnosis of community-acquired pneumonia or healthcare-associated pneumonia (HCAP) and those aged >65 years. The diagnostic criteria for pneumonia were symptoms of the lower respiratory tract (fever, dyspnea, and tachypnea) and new shadows on chest radiography. HCAP was determined when pneumonia developed at a facility for older adults or following medical care such as dialysis. Patients were divided into two groups based on the history of pneumococcal vaccination. This study was conducted according to the Declaration of Helsinki. The ethics committee in Fujisawa City Municipal Hospital (no. F2020054) approved this study, and because of its retrospective nature, the requirement for informed consent was waived.

### 2.2. Baseline Assessment, Definitions, and Follow-Up

Data were collected from electronic medical records, including Eastern Cooperative Oncology Group-Performance Status (ECOG-PS) as an indicator of limitation in life [11]. The time and type (PPSV23 or PCV13) of vaccination against S. *pneumoniae* were not evaluated. The history of vaccination against influenza was also recorded. Respiratory disease included conditions, such as chronic obstructive pulmonary disease (COPD), asthma COPD overlap (ACO), bronchiectasis, and interstitial pneumonia. Laboratory findings were performed on admission. According to current guidelines [12], anti-pseudomonal antibiotic regimens such as *β*-lactams (piperacillin-tazobactam and cefepime), fluoroquinolones (levofloxacin), and carbapenems (meropenem) were administered when pneumonia was determined to be severe or caused by resistant bacteria. In addition, steroids were administered for severe pneumonia or exacerbation of asthma, COPD, or ACO. The severity of pneumonia was classified by A-DROP, a scoring system suitable for assessing the severity of pneumonia, which provided results similar to those of CURB-65 proposed by the British Thoracic Society. A-DROP assessed the following parameters: age (male: ≥70 years; female: ≥75 years), dehydration blood urea nitrogen ≥210 mg/L, respiratory failure defined as oxygen saturation ≤90% or partial pressure of oxygen (PaO_2_) ≤60 mmHg, orientation disturbance, and low blood pressure defined as systolic blood pressure ≤90 mmHg [13, 14]. The pneumonia pathogen was identified from blood and sputum cultures or pneumococcal urinary antigen tests collected from patients on admission. We also compared the prevalence of *S. pneumoniae* in the sputum culture and urinary antigen tests between vaccinated and unvaccinated patients to evaluate the effect of pneumococcal vaccination on bacterial pathogens.

### 2.3. Statistical Analysis

Continuous variables were reported as medians and interquartile ranges (IQR). Significant differences between the two groups were determined using Pearson's *χ*^2^ test or Fisher's exact test, while univariable and multivariable logistic regression analyses were performed to identify associated risk factors for mortality. A *p* value <0.05 was considered significant. The survival of patients was estimated using Kaplan–Meier survival curves. The primary endpoint was in-hospital mortality, and the secondary endpoint was risk factors for mortality. Statistical analyses were performed using JMP Pro 15.0.0 (SAS Institute Inc., Cary, NC, USA). Some incomplete data were omitted from the analysis.

## 3. Results

We included 93 patients with pneumonia in this retrospective study; 38 (41%) had received a pneumococcal vaccination, and 55 (59%) were unvaccinated.  Table 1 shows patient characteristics, demographics, and comorbid conditions' data distribution. There were no significant differences in age, sex, ECOG-PS, and HCAP between vaccinated and unvaccinated patients. However, vaccinated patients received influenza vaccinations more frequently than unvaccinated patients (86.8% vs. 27.3%, *p* < 0.001). There were no differences in hypertension, diabetes, heart disease, kidney disease, neurological disease, and cancer observed between the two groups, but vaccinated patients had a relatively higher prevalence of respiratory disease (68.4% vs. 49.1%, *p*=0.06). Pneumococcal vaccination in vaccinated patients showed that PPV23 was the most frequently used pneumococcal vaccine (*n* = 28; 73.7%). Most patients were vaccinated within 5 years of admission (*n* = 21; 75.0%). Three patients had received PCV13 (7.8%), and one patient had received both PPV23 and PCV13 (Table 1).

**Figure 1:**
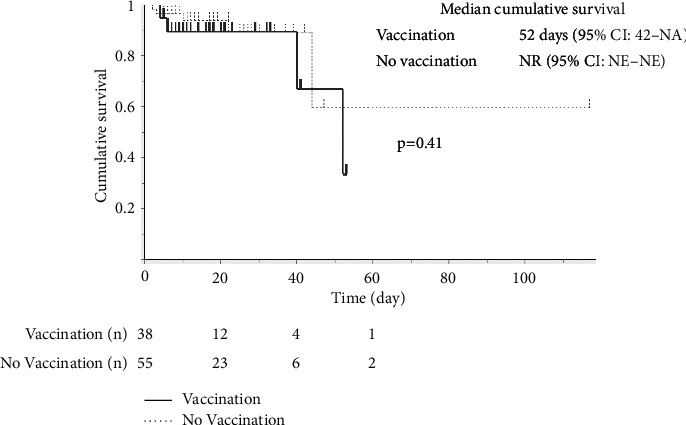
The Kaplan–Meier survival curves for the vaccinated and unvaccinated groups. Six patients in the vaccinated group and five patients in the unvaccinated group did not survive. The survival curves showed no significant difference between the two groups (NA: not available; NR: not reached; NE: not evaluated).

The comparison of laboratory data showed no statistical differences in pneumococcal urinary antigen test results, serum albumin, arterial blood gas (ABG) analysis, or A-DROP scores between the groups (Table 2). The frequency of anti-pseudomonal antibiotics or corticosteroid use was very similar in both groups. Eleven patients did not survive; two of them died of pneumococcal pneumonia. Although the mortality rate was higher in the vaccinated group than in the unvaccinated group (15.8% vs. 9.1%, *p*=0.33), this difference was not significant. Additionally, no statistical differences were seen in the length of hospital stay (12 days vs. 14 days, *p*=0.62) (Table 2). Two patients in the vaccinated group and 10 patients in the unvaccinated group were transferred to another hospital after recovery from pneumonia; therefore, their data have not been shown. Univariate analysis was performed to identify the risk factors for mortality due to pneumonia (Table 3). pH (7.46 vs. 7.38, *p*=0.002) and partial pressure of carbon dioxide (PaCO_2_) of ABG analysis (35.9 mmHg vs. 55.2 mmHg, *p*=0.001), anti-pseudomonal antibiotic use (22.0% vs. 63.6%, *p*=0.006), and A-DROP scores (2 vs. 3, *p*=0.02) were significantly associated with mortality for pneumonia. Multivariable logistic regression identified A-DROP score as an independent risk factor for mortality (odds ratio (OR): 2.64; 95% confidence interval (CI): 1.22–5.72, *p*=0.008). However, vaccination against pneumococcus was not significant (OR: 2.71; 95% CI: 0.667–11.02; *p*=0.16) (Table 4). Respiratory disease was not included in multivariable logistic regression because it was not identified as a risk factor for mortality in the univariate analysis results.  Figure 1 shows the Kaplan–Meier survival curves; six patients in the vaccination group and five patients in the unvaccinated group did not survive. The survival curves between the two groups were not significantly different. The distribution of bacterial species isolated in patients with pneumococcal vaccination was compared to evaluate the impact of vaccination on bacterial etiology among enrolled patients (Table 5).

The prevalence of *S*. *pneumoniae* was 18.4% (*n* = 7) in vaccinated patients and 34.6% (*n* = 19) in unvaccinated patients; however, this was not statistically significant (*p*=0.09). There were no significant differences in bacterial etiologies between the two groups. Blood culture was examined in 70 of 93 patients, but only three patients had bacteremic pneumonia. In addition, there was no IPD in this study.

## 4. Discussion

This retrospective study aimed to investigate the clinical features of pneumococcal pneumonia in older adult patients admitted to the hospital and describe the relationship between pneumonia and pneumococcal vaccination. Our analysis of 93 patients indicated that the risk factors for mortality due to pneumonia were pH and partial pressure of carbon dioxide (PaCO_2_) of ABG analysis, anti-pseudomonal antibiotic use, and A-DROP score. The mortality rate was higher in the vaccinated group than in the unvaccinated group without statistical difference. The current study indicated that 40.1% of enrolled patients received a pneumococcal vaccine. This is similar to the PPV23 vaccine coverage (40.8%) reported in a general population survey of Japanese adults aged >65 years, conducted in 2015 [15]. Other countries have reported pneumococcal vaccination coverage rates of 50–70% [16], suggesting that the vaccination rate for older adult patients in Japan is low despite financial support by the Japanese government. In our study, pneumococcal vaccinated patients have received influenza vaccinations more frequently than the unvaccinated patients. A report from Turkey revealed that health literacy was associated with the higher rates of pneumococcal and influenza vaccinations [17]. This suggests that in addition to promoting pneumococcal and influenza vaccinations, the improvement of health literacy might help to increase vaccination rates.

The preceding study reported that low performance status, hypoalbuminemia, metabolic acidosis, tachypnea, and high urea nitrogen were identified as risk factors of mortality for pneumonia [18]. These were partially consistent with our results. It was suggested that alveolar hypoventilation was related to poor prognosis. Low performance status (*p*=0.08) and hypoalbuminemia (*p*=0.15) tended to be associated with mortality (Table 3). Therefore, it is important to manage and maintain health in daily life before the onset of pneumonia. Proper nutrition has been shown to reduce the prevalence of pneumonia [19].

Table 4 shows that the history of pneumococcal vaccination was related to high mortality (OR: 2.71; 95% CI: 0.667–11.02; *p*=0.16). The patients with respiratory disease in the vaccinated group were more frequent than those in the unvaccinated group (68.4% vs. 49.1%, *p*=0.06). It is said that pneumonia was more severe in patients with COPD and other lung diseases [20]. There could be an unmet difference of background factors that we did not consider, for example, immunocompromised patients. Pneumococcal vaccination is recommended in such immunocompromised patients [21]. In the predisposition, insult, response, and organ dysfunction score, which was the scoring system used to predict mortality among severe CAP, immunocompromise is included [22]. Therefore, the vaccinated group might be more likely to become seriously ill. Originally, the influence of pneumococcal vaccination against pneumonia was controversial. Falkenhorst et al. revealed that PPV23 reduced IPD [5]. A previous study, including nursing home residents, indicated that more nonvaccinated pneumococcal pneumonia patients died compared with vaccinated patients; however, overall mortality from all types of pneumonia was similar in both groups [4].

Our study has several limitations. First, this study was a retrospective review of patients admitted to a single facility over a twelve-month period, and the number of patients was small. Second, the effects of pneumococcal vaccination could not be evaluated because of the small sample size. Third, the duration of time after vaccination might influence its effect because approximately 20% of patients with PPV23 received the pneumococcal vaccine over 5 years previously. The effectiveness of PPV23 appears to wane over a period of 3–5 years [23]. Therefore, the attenuation of the vaccine influences the results.

## 5. Conclusions

Our study suggested that pH and PaCO_2_ of ABG analysis, anti-pseudomonal antibiotic use, and A-DROP scores were the risk factors of mortality for CAP. The history of pneumococcal vaccination was related to increased mortality; however, because of the small sample size, the influence of the vaccination was uncertain.

## Figures and Tables

**Table 1:** Patient characteristics.

	Vaccinated	Unvaccinated	*p* value
	*n* = 38 (%)	*n* = 55 (%)	
*Demographics*			
Age, median (IQR)	81 (74–84)	80 (71–87)	0.94
Males	23 (60.5)	31 (56.3)	0.69
Females	15 (39.5)	24 (43.6)	0.69
PS, median (IQR)	1 (0–2)	1 (0–3)	0.25
HCAP	4 (10.5)	13 (23.6)	0.10
Vaccination against influenza	33 (86.8)	15 (27.3)	<0.001

*Comorbid conditions*			
Hypertension	12 (31.6)	18 (32.8)	0.91
Diabetes	6 (15.8)	7 (12.7)	0.68
Heart disease	15 (39.5)	13 (23.6)	0.1
Kidney disease	3 (7.9)	7 (12.7)	0.46
Neurological disease	6 (15.8)	13 (23.6)	0.36
Cancer	7 (18.4)	5 (9.1)	0.19
Respiratory disease	26 (68.4)	27 (49.1)	0.06

*Types of pneumococcal vaccine*			
PPV23	27 (71.1)		
PCV13	2 (5.2)		
PPV23 + PCV13	1 (2.6)		
Unknown	8 (21.1)		

*Time after PPV23 vaccination*			
<5 years	21 (75.0)		
>5 years	6 (21.4)		
Unknown	1 (2.6)		

IQR: interquartile range; PS: performance status; HCAP: healthcare-associated pneumonia; PPV23: 23-valent pneumococcal polysaccharide vaccine; PCV13: 13-valent pneumococcal conjugate vaccine.

**Table 2:** Comparison of pneumococcal vaccinated and unvaccinated patients.

	Vaccinated	Unvaccinated	*p* value
	*n* = 38 (%)	*n* = 55 (%)	
*Laboratory findings at pneumonia onset*			
Pneumococcal urinary antigen positive	6 (15.8)	18 (32.7)	0.09
Albumin (g/dL), median (IQR)	3.2 (2.9–3.6)	3.0 (2.6–3.6)	0.22

*ABG analysis*			
pH, median (IQR)	7.46 (7.40–7.48)	7.45 (7.39–7.48)	0.49
PaO_2_ (mmHg), median (IQR)	64.0 (56.0–80.4)	69.8 (60.4–81.3)	0.39
PaCO_2_ (mmHg), median (IQR)	35.9 (31.6–43.8)	36.6 (31.4–45.0)	0.91
A-DROP, median (IQR)	2 (2-3)	2 (1–3)	0.56

*Treatment*			
Anti-pseudomonal antibiotic regimens^*∗*^	12 (31.2)	13 (23.6)	0.40
Steroid therapy	12 (31.2)	16 (29.1)	0.80

*Outcome*			
Days of hospitalization, median (IQR)	12 (10–22)	14 (8–28)	0.62
Death	6 (15.8)	5 (9.1)	0.33

IQR: interquartile range; PaO_2_: partial pressure of oxygen; PaCO_2_: partial pressure of carbon dioxide; ABG: arterial blood gas. ^*∗*^Anti-pseudomonal antibiotic regimens: *β*-lactams (piperacillin-tazobactam and cefepime), fluoroquinolones (levofloxacin), or carbapenems (meropenem).

**Table 3:** Univariate analysis of risk factors for mortality.

	Recovered	Not recovered	*p* value
	*n* = 82 (%)	*n* = 11 (%)	
*Demographics*			
Age, median (IQR)	80 (72–84)	82 (77–87)	0.28
Males	46 (56.1)	8 (72.7)	0.29
Females	36 (43.9)	3 (27.3)	0.29
PS, median (IQR)	1 (0–3)	2 (1–3)	0.08
HCAP	16 (19.5)	1 (9.0)	0.46
Respiratory disease	45 (54.9)	8 (72.7)	0.26
Received any pneumococcal vaccination	32 (39.0)	6 (54.5)	0.33
Influenza vaccine	40 (48.8)	8 (72.7)	0.17

*Laboratory findings at pneumonia onset*			
Pneumococcal urinary antigen positive	22 (26.8)	2 (18.2)	0.55
Albumin (g/dL), median (IQR)	3.1 (2.8–3.6)	2.6 (2.2–3.4)	0.15
*ABG*			
pH, median (IQR)	7.46 (7.42–7.48)	7.38 (7.24–7.47)	0.002
PaCO_2_ (mmHg), median (IQR)	35.9 (31.5–42.5)	55.2 (31.6–73.8)	0.002

*Treatment*			
Anti-pseudomonal antibiotic	18 (22.0)	7 (63.6)	0.003
Steroid therapy	25 (30.5)	3 (27.3)	0.83

*Severity of the disease and outcome*			
Days of hospitalization, median (IQR)	14 (10–25)	6 (4–40)	0.74
A-DROP, median (IQR)	2 (1–3)	3 (2–3)	0.02

IQR: interquartile range; PS: performance status; HCAP: healthcare-associated pneumonia; PaCO_2_: partial pressure of carbon dioxide; ABG: arterial blood gas.

**Table 4:** Multivariate analysis of risk factors for mortality.

	OR	95% CI (lower–upper)	*p* value
A-DROP	2.64	1.22–5.72	0.008
Received any pneumococcal vaccination	2.71	0.667–11.02	0.16

OR: odds ratio; CI: confidence interval.

**Table 5:** Distribution of pathogens in pneumococcal vaccinated and unvaccinated patients.

	Vaccinated	Unvaccinated	*p* value
	*n* = 38 (%)	*n* = 55 (%)	
*No. of patients who underwent the test to identify pathogens*			
Sputum culture	34 (89.5)	51 (92.7)	0.58
Pneumococcal urinary antigen positive	34 (89.5)	52 (94.6)	0.36
*Isolated bacteria*			
*Streptococcus pneumoniae* ^*∗*^	7 (18.4)	19 (34.6)	0.09
*Haemophilus influenza*	3 (7.9)	6 (10.9)	0.63
*Klebsiella pneumonia*	3 (7.9)	4 (7.3)	0.91
*Pseudomonas aeruginosa*	9 (23.7)	7 (12.7)	0.17
*MRSA*	2 (5.3)	2 (3.6)	0.70
Normal flora	9 (23.7)	16 (29.1)	0.56

MRSA: methicillin-resistant *Staphylococcus aureus.*^*∗*^Includes pneumococcal urinary antigen positive.

## Data Availability

The data used to support the findings of this study are available from the corresponding author upon request.
